# Auditory Stimulus Detection Partially Depends on Visuospatial Attentional Resources

**DOI:** 10.1177/2041669516688026

**Published:** 2017-01-01

**Authors:** Basil Wahn, Supriya Murali, Scott Sinnett, Peter König

**Affiliations:** Institute of Cognitive Science, University of Osnabrück, Osnabrück, Germany; Department of Psychology, University of Hawai'i at Mānoa, Honolulu, Hawai'i, USA; Institute of Cognitive Science, University of Osnabrück, Osnabrück, Germany; Department of Neurophysiology and Pathophysiology, University Medical Center Hamburg-Eppendorf, Hamburg, Germany

**Keywords:** attentional resources, multisensory processing, vision, audition, multiple object tracking, multisensory integration, load theory

## Abstract

Humans’ ability to detect relevant sensory information while being engaged in a demanding task is crucial in daily life. Yet, limited attentional resources restrict information processing. To date, it is still debated whether there are distinct pools of attentional resources for each sensory modality and to what extent the process of multisensory integration is dependent on attentional resources. We addressed these two questions using a dual task paradigm. Specifically, participants performed a multiple object tracking task and a detection task either separately or simultaneously. In the detection task, participants were required to detect visual, auditory, or audiovisual stimuli at varying stimulus intensities that were adjusted using a staircase procedure. We found that tasks significantly interfered. However, the interference was about 50% lower when tasks were performed in separate sensory modalities than in the same sensory modality, suggesting that attentional resources are partly shared. Moreover, we found that perceptual sensitivities were significantly improved for audiovisual stimuli relative to unisensory stimuli regardless of whether attentional resources were diverted to the multiple object tracking task or not. Overall, the present study supports the view that attentional resource allocation in multisensory processing is task-dependent and suggests that multisensory benefits are not dependent on attentional resources.

## Introduction

The environment provides far more sensory input than can be effectively processed by the human brain. Via a process called “attention” only the information that is currently relevant is selected (Chun, Golomb, & Turk-Browne, 2011; James, 1890; Marois & Ivanoff, 2005). It has been shown that attentional selection is severely limited in resources (i.e., only a limited amount of sensory input can be processed) within each sensory modality (Hillstrom, Shapiro, & Spence, 2002; Potter, Chun, Banks, & Muckenhoupt, 1998; Tremblay, Vachon, & Jones, 2005) and that these limitations depend on the features of stimuli that are processed (Morrone, Denti, & Spinelli, 2002). However, a matter of debate in multisensory research is whether attentional resources for one sensory modality are shared with the resources for another sensory modality or whether there are distinct attentional resources for each sensory modality. The answer to this debate appears to be nuanced, as recent research has argued that the allocation of attentional resources across sensory modalities is task-dependent (Chan & Newell, 2008; Wahn & König, 2016). That is, distinct or shared attentional resources can be found depending on whether tasks involve the discrimination of stimulus attributes (e.g., discriminating pitch or color) or the localization of stimuli (Alais, Morrone, & Burr, 2006; Arrighi, Lunardi, & Burr, 2011; Chan & Newell, 2008; Duncan, Martens, & Ward, 1997; Helbig & Ernst, 2008; Wahn & König, 2015a, 2015b, 2016; Wahn et al., 2016). Specifically, it was found that attentional resources were shared when two spatial tasks are performed in separate sensory modalities (Wahn & König, 2015a, 2015b) while attentional resources were found to be distinct when a spatial task was performed together with a discrimination task (Arrighi et al., 2011; Wahn & König, 2016), and also when two discrimination tasks were performed (Alais et al., 2006; Duncan et al., 1997; Helbig & Ernst, 2008). These findings suggest that spatial tasks rely on shared attentional resources regardless of whether they are performed in the same or separate sensory modalities while the discrimination of stimulus attributes relies on distinct attentional resources for the sensory modalities. However, to date, how this task dependency extends to other task types and other task combinations have yet to be fully explored.

With regard to other types of tasks, such as a detection task, previous research on the phenomenon of “inattentional blindness” has investigated how performing a demanding visual task negatively affects the ability to detect a task-unrelated visual stimulus (Mack & Rock, 1998). However, it was also found that participants’ ability to detect auditory stimuli was lower when performing a demanding visual discrimination task in comparison with performing a less demanding visual discrimination task, providing a cross-modal version of this phenomenon referred to as “inattentional deafness” (Macdonald & Lavie, 2011). Similar results were found in a recent study (Raveh & Lavie, 2015), in which several experiments consistently showed that auditory stimulus detection was affected by task difficulty in a visual search task. As a point of note, a visual search task requires spatial attention as well as object-based attention (Eimer, 2014; Ghorashi, Enns, Klein, & Di Lollo, 2010). That is, humans need to allocate attentional resources to locations in space (i.e., a spatial attention component of the task) and then discriminate whether the attended location is a target or distractor (i.e., an object-based attention component of the task; Eimer, 2014; Ghorashi et al., 2010).

These findings (Macdonald & Lavie, 2011; Raveh & Lavie, 2015) suggest that attentional resources required for visual stimulus discrimination are shared with the resources required for auditory stimulus detection. In an earlier study (Sinnett, Costa, & Soto-Faraco, 2006), auditory as well as visual detection ability was investigated when simultaneously performing a discrimination task in the same or different sensory modality. In this example, when tasks were matched in difficulty, it was found that auditory detection ability was better when a visual discrimination task was performed compared with performing an auditory discrimination task, suggesting that there are also distinct attentional resources for the visual and auditory modalities. Taken together, previous research on detection task performance suggests that auditory stimulus detection only in part relies on visual attentional resources. However, this research has predominantly investigated whether a secondary task involving the discrimination of stimulus attributes (e.g., discriminating target from distractors in a visual search task) affects auditory detection task performance. Accordingly, the extent to which auditory detection task performance relies specifically on visuospatial attentional resources has not been determined. That is, given a visual search task also has an object-based attention component (i.e., discriminating targets from distractors), it has not been explored whether performing a purely *visuospatial* task (i.e., without any requirement to discriminate stimulus features) affects the ability to detect auditory stimuli to the same degree as the ability to detect visual stimuli.

So far, we have addressed the question of how attentional resources are allocated for sensory input that is processed by separate sensory modalities. Yet, sensory processing usually involves several modalities. Depending on how sensory input from multiple sensory modalities is received (i.e., whether it coincides in space or time), it is integrated, resulting in an enhanced perceptual sensitivity (e.g., stimulus features can be processed more efficiently; Ernst & Banks, 2002; Meredith & Stein, 1983; Stein & Stanford, 2008). A matter of ongoing debate is whether the process of multisensory integration is dependent on attentional processes. That is, whether diverting attentional resources away from a task in which stimuli are typically integrated affects the multisensory integration process. Previous studies have both found evidence for that the integration process is affected by attentional processes (Alsius, Möttönen, Sams, Soto-Faraco, & Tiippana, 2014; Alsius, Navarra, Campbell, & Soto-Faraco, 2005; Alsius, Navarra, & Soto-Faraco, 2007; Bertelson, Vroomen, De Gelder, & Driver, 2000; Misselhorn, Daume, Engel, & Friese, 2015; Mozolic, Hugenschmidt, Peiffer, & Laurienti, 2008) or not (Gentile, Guterstam, Brozzoli, & Ehrsson, 2013; Helbig & Ernst, 2008; Wahn & König, 2015a, 2015b, 2016; Zimmer & Macaluso, 2007), indicating several factors that potentially influence how attentional processes affect multisensory integration (Navarra, Alsius, Soto-Faraco, & Spence, 2010; Talsma, 2015). In particular, it has been proposed that the integration of linguistic stimuli (e.g., spoken and written words) is susceptible to attentional processes while the integration of nonlinguistic stimuli (e.g., simple tones and flashes) is not susceptible to attentional processes (Navarra et al., 2010; Wahn & König, 2015a). Similar to the task-dependency with regard to the question of whether there are shared or distinct attentional resources for the sensory modalities, it could be that the integration of stimuli from multiple sensory modalities is also task-dependent. In particular, studies that did not find an effect of attentional processes on multisensory integration for low-level stimuli have predominantly investigated multisensory integration in localization tasks or discrimination tasks. Whether multisensory integration in a detection task is affected when attentional resources are diverted to a spatial task is unknown.

Taken together, the present study aims to investigate two research questions: (a) Are attentional resources shared or distinct when a detection task is performed in combination with a visuospatial task? and (b) Is multisensory integration in a detection task affected when attentional resources are diverted to a simultaneously presented visuospatial task?

We investigated these two research questions using a dual-task paradigm, in which participants performed a visuospatial task (i.e., a multiple object tracking [“MOT”] task; Pylyshyn & Storm, 1988) and a detection task alone or at the same time. In the detection task, participants responded to visual, auditory, or audiovisual targets. In the MOT task, participants tracked a subset of several randomly moving objects. Note that previous research has shown that the MOT task reliably taxes visuospatial attentional resources (Alvarez & Franconeri, 2007; Cavanagh & Alvarez, 2005; Wahn, Ferris, Hairston, & König, 2016). With regard to the first research question, if attentional resources are distinct for the sensory modalities, we hypothesized that performing the MOT task in combination with an auditory detection task should lead to less interference between tasks than performing the MOT task in combination with a visual detection task. Conversely, if attentional resources are shared for the visual and auditory modality, the interference between the MOT task and detection task should be equal regardless of the sensory modality in which the detection task is performed. With regard to the second research question, if visuospatial attentional resources are required for multisensory integration in a detection task, we hypothesized that the benefit from integrating stimuli (i.e., a higher perceptual sensitivity to detect multisensory stimuli than unisensory stimuli) would be lower or disappear completely when performing the MOT task at the same time with the detection task compared with performing the detection task alone. Conversely, if multisensory integration in a detection task is not dependent on visuospatial attentional resources, multisensory integration should not be affected by performing a MOT task simultaneously with the detection task.

## Materials and Methods

### Participants

Twenty students (M = 22.6 years, *SD* = 3.09 years, 14 female) were recruited from the University of Osnabrück to participate in the study in exchange for money or course credits. The study was approved by the ethics committee of the University of Osnabück, and written informed consent was obtained from each participant.

### Experimental Setup

Participants wore headphones (Philips SHL3000WT 00) and were seated in a dark room at a distance of 90 cm in front of a computer screen (BenQ XL2420T, resolution 1920 × 1080, 120 Hz, subtending a visual field of 32.87 × 18.49 visual degrees). We recorded eye movements with a remote eye-tracking system (Eyelink 1000, monocular pupil tracking, 500 Hz sampling rate). To calibrate eye position, we used a five-point grid. The calibration procedure was repeated until the maximum error was below 0.7 visual degrees.

### Staircase Procedure Prior to the Experiment

Prior to starting the experiment, in order to match performance levels in the detection task across sensory modalities, participants’ individual detection thresholds for the visual and auditory detection task were determined using a QUEST (Watson & Pelli, 1983) staircase procedure. In the visual staircase procedure, participants saw a central black dot (0.14 visual degrees) that flashed for 50 ms every second. The participants’ task was to press the “enter” key on the keyboard whenever they detected the flash. A failure to respond was considered a miss. Participants were instructed to press “enter” only when they detected the stimulus. Depending on whether the flash was detected or not, the contrast of the flash relative to the black dot was either lowered or increased using the QUEST staircase procedure, aiming for a 75% detection task performance. The contrast was changed using the RGB color code for the flash and was expressed in a percentage ranging from white (100%, RGB color code: 255, 255, 255) to black (0%, RGB color code: 0, 0, 0). On the basis of pilot data, the initial contrast was set to 30%, and participants received a total of 150 contrast changes. Participants were offered a short break, after every 20th contrast change. Analogously, for the auditory staircase procedure, a “click” sound was received every second via the headphones lasting 50 ms (impulse tone; stereo sound; sampling frequency 44.100 Hz; sound pressure level = 3.8 dBA) that was adjusted downwards or upwards in loudness depending on whether participants detected the sound or not. Matching the number of contrast changes in the staircase procedure for the visual stimuli, a total of 150 sounds were received. The loudness was expressed in a percentage as well and ranged from 0% to 100% (sound pressure level = 3.8 dBA). After the staircase procedure was completed, the last contrast and loudness values of the QUEST procedure were taken as the 75% detection threshold for the visual and auditory detection performance. These thresholds were used as initial thresholds in the QUEST staircase procedures in the detection task conditions that were performed in the actual experiment.

As a point of note, the stimuli in the staircase procedure prior to the experiment were received at a constant rate (i.e., every 1 s). Hence, participants could have predicted the rate of stimulus presentation and pressed the response key every second. Therefore, before the main experiment, the experimenter was instructed to check the responses of the participant whether there is an unusual pattern of responses (i.e., every stimulus was detected toward the end of the QUEST procedure). In case of such a response pattern, the participant was again instructed to only respond when a stimulus was detected, and the initial staircase procedures were repeated.

In the actual experiment, the stimulus onsets were jittered in time, leaving a minimum of 1.7 s and a maximum of 2.5 s between stimulus onsets, and the number of stimuli received in a trial was randomly varied between four and five as well.

### Experimental Procedure

Participants performed the detection task or the MOT task either separately or at the same time in a within-subjects design. We will first describe each task (i.e., the detection task and MOT task) in turn and then describe how they were performed simultaneously.

**Figure 1.**
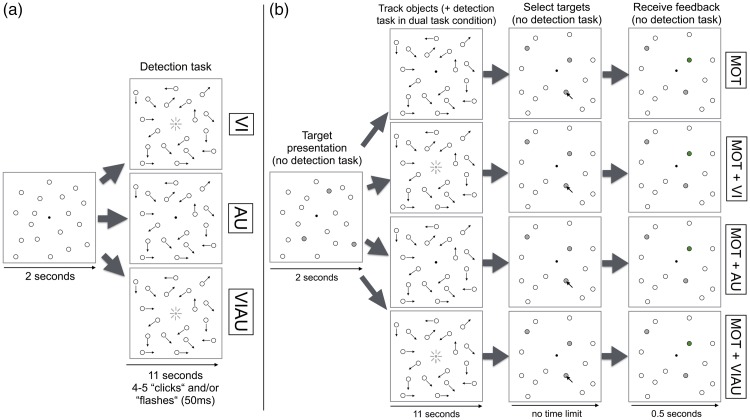
Experimental design overview. (a) Detection task: Depending on condition, participants were required to detect a white flash that always occurred within a black circle in the center of the screen (“VI”, top row), a “click” sound (“AU”, second row), or both the flash and click when presented simultaneously (“VIAU”, third row), by pressing the “enter” key on the keyboard. In all detection task conditions, four to five stimuli were presented per trial that lasted 50 ms and occurred at random time points within a trial. (b) Multiple object tracking task: The trial logic is shown for performing the multiple object tracking task alone (top row), and in combination with the visual (MOT+VI, second row); the auditory (MOT+AU, third row); or the audiovisual (MOT+VIAU, fourth row) detection task.

In the detection task (Figure 1(a)), like in the staircase procedure prior to the experiment, participants either had to detect a white flash that always occurred within a black circle in the center of the screen (“VI” condition) or a “click” sound (“AU” condition) by pressing the “enter” key on the keyboard using their right hand. In a third detection task condition, both, the flash and click were presented simultaneously (“VIAU”) and had to be detected. In all detection task conditions, the stimuli lasted 50 ms and occurred at random time points within a trial, always leaving a minimum of 1.7 s and a maximum of 2.5 s between stimulus onsets. As for the staircases described earlier, if participants did not respond within this time interval (i.e., indicate that they had detected a stimulus), then it was considered a miss. As a point of note, after stimulus onset, we did not set a time limit after which responses would count as false alarms.

For each trial, the number of onsets was randomly varied between four and five. Thus, onsets of stimuli and the number of stimuli were not predictable to the participants. As a dependent measure in the detection task, we used a similar approach as in previous studies investigating attentional resources across sensory modalities (Alais et al., 2006; Arrighi et al., 2011). That is, while participants performed the detection task, stimulus contrast (VI), loudness (AU), or both simultaneously (VIAU) were again adjusted depending on whether participants detected the stimulus or not using a QUEST (Watson & Pelli, 1983) staircase procedure. In particular, as a dependent measure, we estimated for each condition and each experimental block the 75% detection task threshold using the QUEST. Note, at the start of each experimental block, the initial stimulus threshold was the 75% detection threshold determined in the QUEST procedure prior to the experiment for each participant.

In the MOT task (Figure 1(b)), participants had to covertly track a subset of three target objects among 15 distractor objects. Objects were 1.06 visual degrees wide. At the beginning of each trial, the three targets turned gray for 2 s and then became indistinguishable from the other objects and started moving for 11 s. While objects were moving, they bounced off the screen borders and repelled each other. Moreover, the direction and speed of movement was randomly selected with a probability of 1% in each frame (average velocity was 2.57 visual degrees per second and ranged between 1.71 and 3.42 visual degrees per second—the experiment was run at a refresh rate of 100 Hz). Once the movement stopped, participants were asked to determine which objects had originally turned gray (i.e., the targets) using the mouse. Feedback was given at the end of each trial.

In the single task conditions, only one (i.e., VI, AU, or VIAU) of the detection tasks or the MOT task was performed. In the dual task conditions, one of three combinations of the two tasks was performed. Namely, the MOT task was performed in combination with the visual (“MOT+VI”), auditory (“MOT+AU”), or audiovisual (“MOT+VIAU”) detection task. Note, that in all dual task conditions, the detection task was performed only while the objects in the MOT task were moving (i.e., simultaneously). During object motion, participants were instructed to fixate on the center of the screen. Moreover, the objects never moved through the fixation point, thus the visual detection task was spatially separated from the MOT task at all times. To keep all conditions perceptually identical, 18 objects were always displayed regardless of whether participants were required to perform the MOT task or not. That is, while participants performed the detection task alone, objects were still moving.

The experiment had a total of 21 blocks each consisting of 10 trials. After a trial was completed, participants initiated the next trial by pressing the “space” key on the keyboard. Blocks were presented in a pseudorandomized order. Within each block, participants were required to always perform the same condition. The condition was indicated at the start of each block. All seven conditions (VI, AU, VIAU, MOT, MOT+VI, MOT+AU, and MOT+VIAU) were included in each set of seven blocks. We avoided repetitions of a condition in consecutive blocks. After participants completed a set of seven blocks, we offered them an optional break. Prior to starting the experiment, participants were instructed about the procedure of each of the seven conditions separately.

In total, the experiment took about 2 hr. Python was used to program and display the experiment as well as extract the data.

### Data Analysis

Prior to data analysis, we excluded trials in which the participant’s gaze deviated from the center by more than two visual degrees on average (total of 1.13% trials excluded, M = 3.23 visual degrees, *SD* = 1.48 visual degrees of excluded trials).

For the analysis, with regard to the detection task thresholds, we obtained the last threshold value from the QUEST as an estimate of the 75% detection task threshold for each block and calculated the median threshold across all blocks. With regard to the MOT task, we calculated for each trial the fraction of correctly selected targets and took the mean across trials for this measure separately for each condition.

Given that statistical assumptions for parametric tests (i.e., normality) were frequently violated, we used nonparametric tests throughout (e.g., a Wilcoxon-signed rank test) and boxplots to plot the data. All conducted tests are two-sided and as an effect size measure, we report *r* (Rosenthal, 1991).

## Results

### Are Attentional Resources Shared or Distinct for Sensory Modalities When a Detection Task is Performed in Combination With a Visuospatial Task?

**Figure 2.**
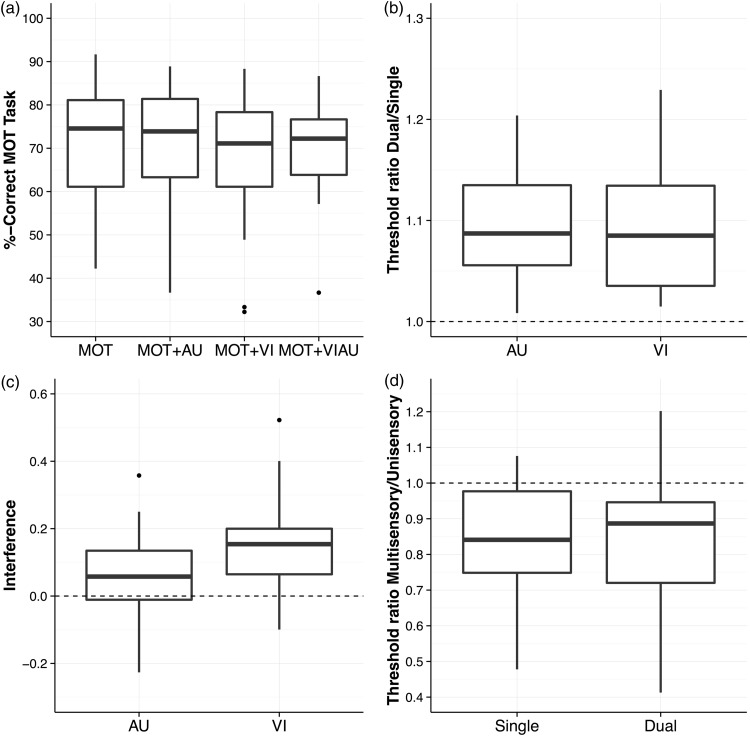
Results overview. (a) Multiple object tracking performance (i.e., fraction correct of target selections) as a function of single task (multiple object tracking) and dual task conditions (MOT+AU, MOT+VI, and MOT+VIAU). (b) Detection task ratios (i.e., threshold in the single task condition divided by the threshold in the dual task condition—values larger than 1 indicate that the multiple object tracking task interfered with the detection task) as a function of detection task conditions (AU and VI). (c) Interference between the multiple object tracking and detection task. For the interference, we first calculated performance ratios for the multiple object tracking performance by dividing the single task condition performance (MOT) separately by each of the dual task conditions (MOT+VI and MOT+AU). A value above 1 indicates that the detection task interfered with performance in the MOT. To have a measure of the overall interference between tasks, we calculated the differences from 1 for the detection task and MOT task ratios and added these differences, separately for each participant and condition. (d) Multisensory detection task ratio (i.e., thresholds in the audiovisual detection task are divided by the unisensory detection task thresholds (VI and AU)). A value below 1 indicates that participants’ perceptual sensitivities to detect stimuli benefits from receiving stimuli from two sensory modalities (i.e., vision and audition) compared with one sensory modality (either vision or audition). Box plots are shown in all panels (1.5 of the interquartile range is used for the whiskers).

We first separately assessed performance in the MOT and detection task. On a descriptive level (Figure 2(a)), it can be seen that MOT performance was negatively affected when performing the MOT task in combination with the VI condition, while performing it in combination with the AU condition did not affect MOT performance. With regard to detection thresholds, to obtain a measure of how much the sensory detection thresholds change between performing the detection task alone or in combination with the MOT task, we divided the thresholds obtained from the QUEST staircase procedure in the dual task conditions (MOT+VI and MOT+AU) by the thresholds obtained from the single task conditions (VI and AU). In this ratio, a value above 1 indicates that detection task thresholds increased (i.e., performance was worse) in the dual task conditions relative to the single task conditions. On a descriptive level, we found that detection task thresholds increased for both types of detection task conditions (Figure 2(b)), suggesting that the MOT task interfered with the ability to detect auditory as well as visual stimuli.

However, to address the question of whether there are separate attentional resources for the visual and auditory modalities, it does not suffice to look at performances of each task separately as participants may devote more attentional resources to one task than the other. As a consequence, it could be that no decrease of performance is visible in one task while a large decrease of performance is visible in the other task, making the interpretation of results difficult. To counteract such an unequal distribution of attentional resources across tasks, we computed an overall score of interference between tasks. To achieve this, we first computed the relative change in MOT performance between the single and dual task conditions by dividing the MOT performance in the single task (MOT) by the performance in the dual task conditions (separately for MOT+VI and MOT+AU). This calculation results in a ratio, in which a measure of above 1 indicates that the detection task interfered with the MOT task. To compute an overall score of interference, we calculated for each participant the difference from 1 for the MOT ratio and the detection task ratio, separately for each condition. The differences from 1 for each ratio (i.e., MOT and detection task ratios) were then summed to have an overall score of interference (for a descriptive overview, see Figure 2(c)). We found that the interference differed significantly from 0 in both cases (VI: *z* = 3.70, *p* < .001, *r* = .83; AU: *z* = 2.58, *p* = .008, *r* = .58), indicating that tasks interfered in both dual task conditions (i.e., MOT+VI and MOT+AU). However, the overall amount of interference between tasks is larger when the MOT task is performed in combination with the visual detection task when compared with the auditory detection task. Given the medians of the interferences (VI: Mdn = 0.16 vs. AU: Mdn = 0.07), the interference is about twice as large in the VI condition than in the AU condition, and a comparison between these conditions is significant (*z* = 2.09, *p* = .036, *r* = .47). Overall, results indicate that attentional resources are only partly shared when an auditory detection task is performed in combination with a MOT task.

To verify that the results given earlier are not due to a speed-accuracy trade-off, we also tested whether response times in the detection task differed between single and dual task conditions. We found no significant difference between the VI and MOT+VI conditions (VI: Mdn = 0.41 s vs. MOT+VI: Mdn = 0.41 s, *z* = 0, *p* = 1, *r* = .00) and AU + MOT+AU conditions (AU: Mdn = 0.46 s vs. MOT+AU: Mdn = 0.46 s, *z* = 0.75, *p* = .475, *r* = .17), suggesting that there is no speed-accuracy trade-off between the single and dual task conditions. That is, participants did not achieve a higher detection sensitivity in the single task condition by taking longer to respond in the single task conditions compared with the dual task conditions.

As an additional analysis, we also tested how performing the MOT task and detection task at the same time affected participants’ ability to fixate on the center of the screen. For this purpose, we tested how the average deviations in eye fixation (measured in visual degrees) from the screen center differed between single and dual task conditions. With regard to how eye fixations in the MOT task were affected by the additionally performed detection task, we found that the average deviations did not significantly differ between single and dual task conditions when the VI detection task (MOT: Mdn = 0.98 visual degrees vs. MOT + VI: Mdn = 0.92 visual degrees, *z* = 1.05, *p* = .312, *r* = .23) or AU detection task (MOT: Mdn = 0.98 visual degrees vs. MOT+AU: Mdn = 0.93 visual degrees, *z* = 0.45, *p* = .674, *r* = .10) was additionally performed. With regard to how fixations were affected in the detection task when additionally performing the MOT task, we did not find any significant differences between single and dual task conditions as well (VI: Mdn = 0.85 visual degrees vs. MOT + VI: Mdn = 0.92 visual degrees, *z* = 0.52, *p* = .622, *r* = .12; AU: Mdn = 0.80 visual degrees vs. MOT + AU: Mdn = 0.93 visual degrees, *z* = 0.93, *p* = .368, *r* = .21).

### Is Multisensory Integration in a Detection Task Affected When Attentional Resources Are Diverted to a Visuospatial Task?

With regard to the second research question, we first tested whether there is a multisensory benefit in the single and dual task condition. For this purpose, for each participant, we first divided the visual contrast threshold in the VIAU condition by the visual contrast in the VI condition and the auditory loudness threshold in the VIAU condition by the auditory loudness threshold in the AU condition. We then averaged across these two ratios for each participant to have an overall estimate how the sensitivity improves in the multisensory condition relative to the unimodal conditions (i.e., values below 1 would indicate a multisensory benefit). This procedure was also repeated for the dual task conditions. For a descriptive overview of these ratios, see Figure 2(d).

We found a multisensory benefit in both conditions (single: *z* = 3.47, *p* < .001, *r* = .78; dual: *z* = 3.21, *p* < .001, *r* = .72), indicating that participants’ perceptual sensitivities to detect stimuli increased when receiving audiovisual stimuli compared with receiving only visual or auditory stimuli. To test whether this multisensory benefit is higher in the single than in the dual task condition, we then compared whether these ratios significantly differed between these two conditions. We found no significant difference between these conditions (*z* = 0.30, *p* = .784, *r* = .07), suggesting that the magnitude of the multisensory benefit does not depend on the available visuospatial attentional resources.

We also repeated this analysis for a more conservatively estimated ratio to assess the multisensory benefit. In particular, we took the lowest estimated unisensory threshold for each participant (instead of the median one—as described in the “Methods of data analysis” section earlier) and then divided the multisensory threshold by these unisensory estimates. With this more conservatively estimated ratio, we found a similar pattern of results: A significant multisensory benefit for the single task condition (Mdn = 0.91, *z* = 2.02, *p* = .045, *r* = .45) and a trend toward significance for the dual task condition (Mdn = 0.94, *z* = 1.83, *p* = .070, *r* = .41). Comparing ratios between these conditions again yielded no significant difference (*z* = 0.22, *p* = .841, *r* = .05).

**Figure 3.**
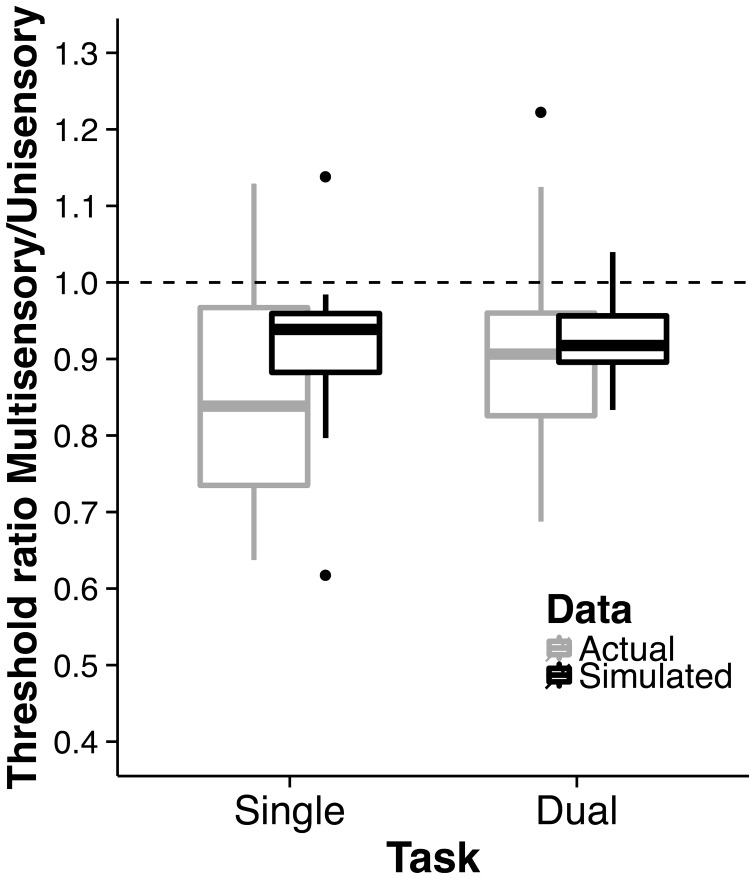
Multisensory benefit. Shown are multisensory detection task ratios (based on 75% thresholds from fitted psychometric functions) as a function of single and dual task conditions—separately for the actual data and simulated data; 1.5 of the interquartile range is used for the whiskers in the box plots.

In addition, we also tested whether the multisensory benefit found earlier is due to the process of multisensory integration or could be alternatively explained by probability summation. In the case of probability summation, participants’ increased sensitivity in the bimodal condition could simply be due to the fact that they received two stimuli compared with one of the them in the unimodal conditions, resulting in a higher sensitivity. In the case of multisensory integration, the bimodal sensitivity is assumed to be higher than estimated by probability summation. For this purpose, we extracted the unimodal staircase sequences for the VI and AU condition and took the maximum of the VI and AU responses for each presented stimulus. For instance, for a presented stimulus, if the participant would not have detected the visual stimulus but did detect the auditory stimulus, then the (maximum) performance would be that the participant still detected the stimulus. On the basis of these calculated responses, using logistic regression, we fitted psychometric functions to the visual contrast and loudness values, respectively, and extracted the 75% thresholds of these functions. We repeated this procedure also for the MOT+VI and MOT+AU conditions. In addition to fitting psychometric functions to these simulated performances, we also fitted psychometric functions to the staircase sequences of all other conditions and extracted 75% thresholds. In line with the analysis earlier, we computed ratios between the unimodal thresholds and bimodal thresholds. In addition, we also computed these ratios for the simulated bimodal thresholds. A descriptive overview is shown in Figure 3. We found that the ratios for the actual data did not significantly differ from the simulated data (Single: *z* = −1.46, *p* = .154, *r* = .33; Dual: *z* = 1.16, *p* = .261, *r* = .26). Given these results, we cannot exclude the possibility that the multisensory benefit is due to probability summation.

Overall, results suggest that multisensory benefits (i.e., a higher sensitivity for bimodal stimuli compared with unimodal stimuli) in a detection task are not affected by simultaneously performing a visuospatial task.

## Discussion

In the present study, we examined how attentional processes and multisensory processing are interrelated. Specifically, we investigated two research questions: (a) Are attentional resources shared or distinct for the sensory modalities when a detection task is performed in combination with a visuospatial task? and (b) Is multisensory integration in a detection task affected when attentional resources are diverted to a visuospatial task?

With regard to the first question, we found that auditory stimulus detection is in part dependent on visuospatial attentional resources as the MOT task did interfere with auditory detection task performance. However, results also suggest that the MOT task interfered to a greater extent with performance on the visual detection task (i.e., about twice as much), thereby suggesting that there are at least partly shared attentional resources for vision and audition for the present task combination.

These findings dovetail with previous studies that have investigated the question of shared or distinct resources for these two sensory modalities using either detection or discrimination tasks (Alais et al., 2006; Sinnett et al., 2006). Specifically, these previous findings also indicated distinct attentional resources when two discrimination tasks or a detection task in combination with a discrimination task were performed in separate sensory modalities. The present study extends these findings by showing that attentional resources are partly shared for vision and audition when a detection task is performed in combination with a task that taxes visuospatial attentional resources.

From a neurophysiological perspective, the present findings align with previous studies that have shown that there are more attentional resources available across sensory modalities than within a sensory modality (Finoia et al., 2015; Haroush, Deouell, & Hochstein, 2011; Rees, Frith, & Lavie, 2001). For instance, Rees et al. (2001) found neurophysiological and behavioral evidence that visual motion processing was unaffected when performing either an easy or difficult auditory detection task. Similar to the present study, participants were required to process visual motion information while simultaneously performing an auditory detection task. However, in contrast to this previous research, participants in the present investigation did not only passively view concurrent visuospatial information while performing an auditory task but had to actively perform two tasks in two separate sensory modalities. Nonetheless, we found less interference between tasks when tasks were performed in separate sensory modalities than within the same sensory modality, suggesting that future neurophysiological studies that require participants to actively and simultaneously perform two tasks (i.e., an auditory detection and a visuospatial task) would also find neural correlates of distinct attentional resources for the visual and auditory modalities.

With regard to the second research question, we found that the multisensory benefit (i.e., a better detection task performance in multisensory conditions than in unisensory conditions) is robust against diverting visuospatial resources to a secondary task. However, we cannot exclude the possibility that this benefit is due to probability summation rather than the process of multisensory integration. These findings are in line with previous studies arguing that for low-level stimuli (e.g., such as tones) multisensory benefits are not dependent on attentional resources (Wahn & König, 2015a, b, 2016; Zimmer & Macaluso, 2007).

A number of potential confounds need to be addressed. Namely, one might argue that the benefit of performing tasks in separate sensory modalities (i.e., vision and audition) in comparison to performing them in the same sensory modality (i.e., vision) is due to oculomotor limitations. However, it should be noted that participants were not required to repeatedly switch their gaze between tasks to perform the visual detection task and the MOT task at the same time—only distributing attentional resources between tasks were required as participants were instructed to fixate their gaze at the center of the screen.

However, a confound that cannot be fully discounted is the requirement to continuously press a key in the detection task while this is not required in the MOT task. This motor component in the detection task may cause interference between tasks that is independent of the sensory modalities in which they are performed. Previous research investigating the psychological refractory period indeed has shown that attentional resources for performing actions in tasks are independent of the sensory modalities in which they are performed (Dux, Ivanoff, Asplund, & Marois, 2006; Marois & Ivanoff, 2005; Pashler, 1994). Therefore, we cannot discount the possibility that the observed interference between the MOT task and the auditory detection task may be due to this motor component in the detection task. Given this possibility and if tasks without any motor component would have been performed, attentional resources for vision and audition could be completely distinct for the present task combination. Future studies could address this point by using a dual task design that does not require participants to perform motor actions when performing two tasks at the same time.

Another point of note related the motor component in the task is that participants could have adopted a strategy in which they estimate the rate of stimulus onsets in the detection task and continuously press the key regardless of the contrast or loudness of the stimuli, respectively. We did jitter the onsets and varied the number of the stimuli to counteract such a strategy. However, we cannot fully exclude the possibility that participants nonetheless adopted such a strategy—also due to the fact that we did not include the possibility of committing false alarms in our experimental design. However, this limitation applies to all experimental conditions that included the detection task. That is, systematic differences between conditions cannot be explained due to such a response strategy. Future studies could counteract this limitation in the design by setting a time limit for responses after stimulus onset (e.g., 1 s) and consider responses after the time limit as false alarms.

As an additional point of note, previous findings have indicated that when performing two spatial tasks in separate sensory modalities that attentional resources are completely overlapping, suggesting that spatial processing is modality-independent (Wahn & König, 2015a, b). Notably, the present detection task did not include any spatial uncertainty. That is, the location where the visual or auditory stimuli would appear was kept constant throughout the experiment. Future studies could investigate how the allocation of attentional resources across sensory modalities systematically changes with the spatial uncertainty where a stimulus in a detection task appears.

With regard to the second research question, an alternative explanation for finding no dependency of multisensory benefit on visuospatial attentional resources could be that the amount of attentional resources diverted from the detection task was too low. In particular, it could be that a more difficult visuospatial task (e.g., a MOT task in which five targets instead of three targets need to be tracked) could have affected the multisensory benefit. However, previous studies using a similar design (i.e., also a MOT task with three targets) in which the overall interference between tasks was higher also found that the increased demand of visuospatial attentional resources did not affect the multisensory benefit (Wahn & König, 2015a, b).

Another alternative account of our findings would be that the observed improvement in detection task performance when receiving audiovisual stimuli is not due to the process of multisensory integration but could instead be due to an alternation strategy. That is, participants could have chosen to always respond to the stimulus in the sensory modality that they can detect more easily. However, this account seems unlikely given that detection thresholds were matched to equal performance levels for each sensory modality prior to the experiment. Moreover, findings related to such an alternation strategy typically result in faster reaction times (Diederich & Colonius, 2004; Sinnett, Soto-Faraco, & Spence, 2008; Vroomen & Gelder, 2000). In the present study, however, we found improved perceptual sensitivities—a perceptual benefit associated with the process of multisensory integration (Ernst & Banks, 2002; Helbig & Ernst, 2008; Stein & Stanford, 2008; Wahn & König, 2016). That is, participants were able to detect audiovisual stimuli at lower stimulus intensities than in unisensory conditions. Yet, the improved sensitivities in the audiovisual condition did not surpass our simulated audiovisual sensitivities which were estimated under the assumption that the auditory and visual stimuli are processed independently. Hence, we cannot exclude that the visual and auditory stimuli were not truly integrated in the audiovisual detection task. A possible reason why stimuli might not have been integrated could be that they were not received from the same spatial location (i.e., auditory stimuli were received via headphones, and visual stimuli were presented on the screen). A future study could address this point by providing audiovisual stimuli from the same spatial location, using loudspeakers instead of headphones.

Overall, the findings of the present study suggest that attentional resources required for auditory stimulus detection are partly shared with the attentional resources required for visuospatial processing. Even though visuospatial processing resources are required for auditory as well as visual stimulus detection, multisensory benefits for audiovisual stimuli in a detection task were not affected by diverting visuospatial attentional resources away from the detection task.

With regard to future studies, relevant investigation could explore the extent spatial attentional demands in a different sensory modality than vision interfere with stimulus detection. That is, in the present study, we investigated how increasing the demand of visuospatial attentional resources affected auditory stimulus detection. A future study could investigate to what extent a spatial auditory task would interfere with visual stimulus detection performance. Given that humans tend to have a processing preference for visual sensory input (e.g., see the “Colavita Effect” Colavita, 1974; Hartcher-O’Brien, Gallace, Krings, Koppen, & Spence, 2008; Hartcher-O’Brien, Levitan, & Spence, 2010; Hecht & Reiner, 2009; Koppen, Levitan, & Spence, 2009; Sinnett, Spence, & Soto-Faraco, 2007; Spence, Parise, & Chen, 2012), it is possible that visual stimulus detection might be unaffected when an auditory spatial attention task is performed simultaneously. Moreover, given that this visual preference effect is dependent on task demands (Chandra, Robinson, & Sinnett, 2011; Robinson, Ahmar, & Sloutsky, 2010; Robinson, Chandra, & Sinnett, 2016), other task combinations (e.g., involving temporal order judgements) could yet yield a different pattern of results. For instance, performance in an auditory temporal order judgment task may be unaffected by visual attentional load while the reverse may not the case.

## References

[bibr1-2041669516688026] Alais D. Morrone C. Burr D. (2006) Separate attentional resources for vision and audition. Proceedings of the Royal Society B: Biological Sciences 273: 1339–1345. 16777721 10.1098/rspb.2005.3420 PMC1560294

[bibr2-2041669516688026] Alsius, A., Möttönen, R., Sams, M. E., Soto-Faraco, S., & Tiippana, K. (2014). Effect of attentional load on audiovisual speech perception: evidence from erps. *Frontiers in Psychology*, *5*, 1–9. 10.3389/fpsyg.2014.00727 PMC4097954 25076922

[bibr3-2041669516688026] Alsius A. Navarra J. Campbell R. Soto-Faraco S. (2005) Audiovisual integration of speech falters under high attention demands. Current Biology 15: 839–843. 15886102 10.1016/j.cub.2005.03.046

[bibr4-2041669516688026] Alsius A. Navarra J. Soto-Faraco S. (2007) Attention to touch weakens audiovisual speech integration. Experimental Brain Research 183: 399–404. 17899043 10.1007/s00221-007-1110-1

[bibr5-2041669516688026] Alvarez G. A. Franconeri S. L. (2007) How many objects can you track?: Evidence for a resource-limited attentive tracking mechanism. Journal of Vision 7: 14. 10.1167/7.13.14 17997642

[bibr6-2041669516688026] Arrighi, R., Lunardi, R., & Burr, D. (2011). Vision and audition do not share attentional resources in sustained tasks. *Frontiers in Psychology*, *2*, 1–4. 10.3389/fpsyg.2011.00056 PMC3110771 21734893

[bibr7-2041669516688026] Bertelson P. Vroomen J. De Gelder B. Driver J. (2000) The ventriloquist effect does not depend on the direction of deliberate visual attention. Perception & Psychophysics 62: 321–332. 10723211 10.3758/bf03205552

[bibr8-2041669516688026] Cavanagh P. Alvarez G. A. (2005) Tracking multiple targets with multifocal attention. Trends in Cognitive Sciences 9: 349–354. 15953754 10.1016/j.tics.2005.05.009

[bibr9-2041669516688026] Chan J. S. Newell F. N. (2008) Behavioral evidence for task-dependent “what” versus “where” processing within and across modalities. Perception & Psychophysics 70: 36–49. 18306959 10.3758/pp.70.1.36

[bibr10-2041669516688026] Chandra, M., Robinson, C. W., & Sinnett, S. (2011). Coexistence of multiple modal dominances. *Proceedings of the 33rd Annual Conference of the Cognitive Science Society, Cognitive Science Society Austin*, TX.

[bibr11-2041669516688026] Chun M. M. Golomb J. D. Turk-Browne N. B. (2011) A taxonomy of external and internal attention. Annual Review of Psychology 62: 73–101. 10.1146/annurev.psych.093008.100427 19575619

[bibr12-2041669516688026] Colavita F. B. (1974) Human sensory dominance. Perception & Psychophysics 16: 409–412.

[bibr13-2041669516688026] Diederich A. Colonius H. (2004) Bimodal and trimodal multisensory enhancement: Effects of stimulus onset and intensity on reaction time. Perception & Psychophysics 66: 1388–1404. 15813202 10.3758/bf03195006

[bibr14-2041669516688026] Duncan J. Martens S. Ward R. (1997) Restricted attentional capacity within but not between sensory modalities. Nature 397: 808–810. 10.1038/42947 9194561

[bibr15-2041669516688026] Dux P. E. Ivanoff J. Asplund C. L. Marois R. (2006) Isolation of a central bottleneck of information processing with time-resolved fmri. Neuron 52: 1109–1120. 17178412 10.1016/j.neuron.2006.11.009 PMC2527865

[bibr16-2041669516688026] Eimer M. (2014) The neural basis of attentional control in visual search. Trends in Cognitive Sciences 18: 526–535. 24930047 10.1016/j.tics.2014.05.005

[bibr17-2041669516688026] Ernst M. O. Banks M. S. (2002) Humans integrate visual and haptic information in a statistically optimal fashion. Nature 415: 429–433. 11807554 10.1038/415429a

[bibr18-2041669516688026] Finoia P. Mitchell D. J. Hauk O. Beste C. Pizzella V. Duncan J. (2015) Concurrent brain responses to separate auditory and visual targets. Journal of Neurophysiology 114: 1239–1247. 26084914 10.1152/jn.01050.2014 PMC4540000

[bibr19-2041669516688026] Gentile G. Guterstam A. Brozzoli C. Ehrsson H. H. (2013) Disintegration of multisensory signals from the real hand reduces default limb self-attribution: An fmri study. The Journal of Neuroscience 33: 13350–13366. 23946393 10.1523/JNEUROSCI.1363-13.2013 PMC3742923

[bibr20-2041669516688026] Ghorashi S. Enns J. T. Klein R. M. Di Lollo V. (2010) Spatial selection and target identification are separable processes in visual search. Journal of Vision 10: 7. 10.1167/10.3.7 20377284

[bibr21-2041669516688026] Haroush K. Deouell L. Y. Hochstein S. (2011) Hearing while blinking: Multisensory attentional blink revisited. The Journal of Neuroscience 31: 922–927. 21248117 10.1523/JNEUROSCI.0420-10.2011 PMC6632914

[bibr22-2041669516688026] Hartcher-O’Brien J. Gallace A. Krings B. Koppen C. Spence C. (2008) When vision ‘extinguishes’ touch in neurologically-normal people: Extending the colavita visual dominance effect. Experimental Brain Research 186: 643–658. 18301885 10.1007/s00221-008-1272-5

[bibr23-2041669516688026] Hartcher-O’Brien J. Levitan C. Spence C. (2010) Out-of-touch: Does vision dominate over touch when it occurs off the body. Brain Research 1362: 48–55. 20850418 10.1016/j.brainres.2010.09.036

[bibr24-2041669516688026] Hecht D. Reiner M. (2009) Sensory dominance in combinations of audio, visual and haptic stimuli. Experimental Brain Research 193: 307–314. 18985327 10.1007/s00221-008-1626-z

[bibr25-2041669516688026] Helbig H. B. Ernst M. O. (2008) Visual-haptic cue weighting is independent of modality-specific attention. Journal of Vision 8: 21. 10.1167/8.1.21 18318624

[bibr26-2041669516688026] Hillstrom A. P. Shapiro K. L. Spence C. (2002) Attentional limitations in processing sequentially presented vibrotactile targets. Perception & Psychophysics 64: 1068–1082. 12489662 10.3758/bf03194757

[bibr27-2041669516688026] James W. (1890) The principles of psychology, Cambridge, MA: Harvard University Press.

[bibr28-2041669516688026] Koppen C. Levitan C. A. Spence C. (2009) A signal detection study of the colavita visual dominance effect. Experimental Brain Research 196: 353–360. 19488743 10.1007/s00221-009-1853-y

[bibr29-2041669516688026] Macdonald J. S. Lavie N. (2011) Visual perceptual load induces inattentional deafness. Attention, Perception, & Psychophysics 73: 1780–1789. 10.3758/s13414-011-0144-4 PMC3152714 21611856

[bibr30-2041669516688026] Mack A. Rock I. (1998) Inattentional blindness Vol. 33: Cambridge, MA: MIT Press.

[bibr31-2041669516688026] Marois R. Ivanoff J. (2005) Capacity limits of information processing in the brain. Trends in Cognitive Sciences 9: 296–305. 15925809 10.1016/j.tics.2005.04.010

[bibr32-2041669516688026] Meredith M. A. Stein B. E. (1983) Interactions among converging sensory inputs in the superior colliculus. Science 221: 389–391. 6867718 10.1126/science.6867718

[bibr33-2041669516688026] Misselhorn, J., Daume, J., Engel, A. K., & Friese, U. (2015). A matter of attention: Crossmodal congruence enhances and impairs performance in a novel trimodal matching paradigm. *Neuropsychologia*, *88*, 113–122. 10.1016/j.neuropsychologia.2015.07.022 26209356

[bibr34-2041669516688026] Morrone M. C. Denti V. Spinelli D. (2002) Color and luminance contrasts attract independent attention”. Current Biology 12: 1134–1137. 12121622 10.1016/s0960-9822(02)00921-1

[bibr35-2041669516688026] Mozolic J. L. Hugenschmidt C. E. Peiffer A. M. Laurienti P. J. (2008) Modality-specific selective attention attenuates multisensory integration. Experimental Brain Research 184: 39–52. 17684735 10.1007/s00221-007-1080-3

[bibr36-2041669516688026] Navarra J. Alsius A. Soto-Faraco S. Spence C. (2010) Assessing the role of attention in the audiovisual integration of speech. Information Fusion 11: 4–11.

[bibr37-2041669516688026] Pashler H. (1994) Dual-task interference in simple tasks: Data and theory. Psychological Bulletin 116: 220. 7972591 10.1037/0033-2909.116.2.220

[bibr38-2041669516688026] Potter M. C. Chun M. M. Banks B. S. Muckenhoupt M. (1998) Two attentional deficits in serial target search: The visual attentional blink and an amodal task-switch deficit. Journal of Experimental Psychology: Learning, Memory, and Cognition 24: 979–992. 10.1037//0278-7393.24.4.979 9699304

[bibr39-2041669516688026] Pylyshyn Z. W. Storm R. W. (1988) Tracking multiple independent targets: Evidence for a parallel tracking mechanism”. Spatial Vision 3: 179–197. 3153671 10.1163/156856888x00122

[bibr40-2041669516688026] Raveh D. Lavie N. (2015) Load-induced inattentional deafness. Attention, Perception, & Psychophysics 77: 483–492. 10.3758/s13414-014-0776-2 PMC4677383 25287617

[bibr41-2041669516688026] Rees G. Frith C. Lavie N. (2001) Processing of irrelevant visual motion during performance of an auditory attention task. Neuropsychologia 39: 937–949. 11516446 10.1016/s0028-3932(01)00016-1

[bibr42-2041669516688026] Robinson, C. W., Ahmar, N., & Sloutsky, V. M. (2010). Evidence for auditory dominance in a passive oddball task. *Proceedings of the 32nd Annual Conference of the Cognitive Science Society, Cognitive Science Society Austin*, TX.

[bibr43-2041669516688026] Robinson C. W. Chandra M. Sinnett S. (2016) Existence of competing modality dominances. Attention, Perception, & Psychophysics 78: 1104–1114. 10.3758/s13414-016-1061-3 26832916

[bibr44-2041669516688026] Rosenthal, R. (1991). *Meta-analytic procedures for social research* (Vol. 6). Newbury Park, CA: SAGE Publications.

[bibr45-2041669516688026] Sinnett S. Costa A. Soto-Faraco S. (2006) Manipulating in attentional blindness within and across sensory modalities. The Quarterly Journal of Experimental Psychology 59: 1425–1442. 16846969 10.1080/17470210500298948

[bibr46-2041669516688026] Sinnett S. Soto-Faraco S. Spence C. (2008) The co-occurrence of multisensory competition and facilitation”. Acta Psychologica 128: 153–161. 18207117 10.1016/j.actpsy.2007.12.002

[bibr47-2041669516688026] Sinnett S. Spence C. Soto-Faraco S. (2007) Visual dominance and attention: The colavita effect revisited. Perception & Psychophysics 69: 673–686. 17929691 10.3758/bf03193770

[bibr48-2041669516688026] Spence C. Parise C. Chen Y. C. (2012) The colavita visual dominance effect. The Neural Bases of Multisensory Processes 1: 523–550.

[bibr49-2041669516688026] Stein B. E. Stanford T. R. (2008) Multisensory integration: Current issues from the perspective of the single neuron. Nature Reviews Neuroscience 9: 255–266. 18354398 10.1038/nrn2331

[bibr50-2041669516688026] Talsma, D. (2015). Predictive coding and multisensory integration: An attentional account of the multisensory mind. *Frontiers in Integrative Neuroscience, 9*, 1–13. 10.3389/fnint.2015.00019 PMC4374459 25859192

[bibr51-2041669516688026] Tremblay S. Vachon F. Jones D. M. (2005) Attentional and perceptual sources of the auditory attentional blink. Perception & Psychophysics 67: 195–208. 15971684 10.3758/bf03206484

[bibr52-2041669516688026] Vroomen J. Gelder B. D. (2000) Sound enhances visual perception: Cross-modal effects of auditory organization on vision. Journal of Experimental Psychology: Human Perception and Performance 26: 1583. 11039486 10.1037//0096-1523.26.5.1583

[bibr53-2041669516688026] Wahn, B., Ferris, D. P., Hairston, W. D., & König, P. (2016). Pupil sizes scale with attentional load and task experience in a multiple object tracking task. *PLoS One*, *11*, 1–15. 10.1371/journal.pone.0168087 PMC5157994 27977762

[bibr54-2041669516688026] Wahn, B., & König, P. (2015a). Audition and vision share spatial attentional resources, yet attentional load does not disrupt audiovisual integration. *Frontiers in Psychology, 6*, 1–12. 10.3389/fpsyg.2015.01084 PMC4518141 26284008

[bibr55-2041669516688026] Wahn B. König P. (2015b) Vision and haptics share spatial attentional resources and visuotactile integration is not affected by high attentional load. Multisensory Research 28: 371–392. 26288905 10.1163/22134808-00002482

[bibr56-2041669516688026] Wahn, B., & König, P. (2016). Attentional resource allocation in visuotactile processing depends on the task, but optimal visuotactile integration does not depend on attentional resources. *Frontiers in Integrative Neuroscience, 10*, 1–13. 10.3389/fnint.2016.00013 PMC4781873 27013994

[bibr57-2041669516688026] Wahn B. Schwandt J. Krüger M. Crafa D. Nunnendorf V. König P. (2016) Multisensory teamwork: Using a tactile or an auditory display to exchange gaze information improves performance in joint visual search. Ergonomics 59: 781–795. 26587687 10.1080/00140139.2015.1099742

[bibr58-2041669516688026] Watson A. B. Pelli D. G. (1983) Quest: A bayesian adaptive psychometric method. Perception & Psychophysics 33: 113–120. 6844102 10.3758/bf03202828

[bibr59-2041669516688026] Zimmer U. Macaluso E. (2007) Processing of multisensory spatial congruency can be dissociated from working memory and visuo-spatial attention. European Journal of Neuroscience 26: 1681–1691. 17880400 10.1111/j.1460-9568.2007.05784.x

